# Consequential late effects after radiotherapy for prostate cancer - a prospective longitudinal quality of life study

**DOI:** 10.1186/1748-717X-5-27

**Published:** 2010-04-08

**Authors:** Michael Pinkawa, Richard Holy, Marc D Piroth, Karin Fischedick, Sandra Schaar, Dalma Székely-Orbán, Michael J Eble

**Affiliations:** 1 Department of Radiation Oncology, RWTH Aachen University, Pauwelsstrasse 30, 52072 Aachen, Germany

## Abstract

**Background:**

To answer the question if and to which extent acute symptoms at the end and/or several weeks after radiotherapy can predict adverse urinary and gastrointestinal long-term quality of life (QoL).

**Methods:**

A group of 298 patients has been surveyed prospectively before (time A), at the last day (B), two months after (C) and >one year after (D) radiotherapy using a validated questionnaire (Expanded Prostate Cancer Index Composite). A subgroup of 10% with the greatest urinary/bowel bother score decrease at time D was defined as patients with adverse long-term QoL.

**Results:**

Subgroup and correlation analyses could demonstrate a strong dependence of urinary/bowel QoL after radiotherapy on urinary/bowel QoL before radiotherapy. In contrast to absolute scores, QoL score changes (relative to baseline scores) did not correlate with pretreatment scores. Long-term changes could be well predicted by acute changes. Patients reporting great/moderate bother with urinary/bowel problems at time C reported to have great/moderate bother at time D in ≥ 50%, respectively. In a multivariate analysis of factors for adverse long-term urinary and bowel QoL, score changes at time C were found to be independent predictors, respectively. Additionally, QoL changes at time B were independently predictive for adverse long-term bowel QoL.

**Conclusions:**

Consequential late effects play a major role after radiotherapy for prostate cancer. Patients with greater and particularly longer non-healing acute toxicity are candidates for closer follow-up and possible prophylactic actions to reduce a high probability of long-term problems.

## Background

External beam radiotherapy is a well established curative treatment for localized prostate cancer [1]. Acute and late toxicity rates after radiotherapy can be considerable and have been subject of many studies. Dose-volume effect relationships have been described extensively [2-6]. Dose escalation studies support the benefit of a dose escalation to total doses approaching 80 Gy concerning the biochemical tumour control or disease-specific survival [7-9]. Subgroups of patients - especially patients with low initial PSA levels <10 ng/ml [8] or <15 ng/ml [7] or low-risk patients [9] - have not been convincingly shown to benefit from total doses >70 Gy. Dose escalation was also associated with a significant increase in late gastrointestinal toxicity [9,10].

In the early years of radiotherapy, the "skin erythema dose" was used for the definition of tolerable doses. During subsequent years, it was realized that no relationship between acute reactions to radiation exposure and late sequelae in other organs and tissues could be established in the majority of patients. More aggressive radiotherapy protocols can result in aggravation, i.e. an increase in severity and duration, of acute radiation effects. Particularly in those organ systems in which a barrier against mechanical and/or chemical stress is established by the acutely responding component - a non-healing acute response can directly progress into a late effect. This phenomenon has been termed a consequential late effect [11].

Consequential late effects have also been reported for prostate cancer patients [12,13]. However, though quality of life (QoL) issues are increasingly addressed in the literature [14-17], the impact of acute on late QoL changes has not been analyzed before.

The aim of this study was to answer the question if and to which extent acute symptoms at the end and/or several weeks after radiotherapy can predict adverse urinary and gastrointestinal long-term quality of life (QoL). Patients responded to a QoL questionnaire before, at the last day, two months (median time) after and more than one year after treatment. QoL score changes in the urinary and bowel domain relative to the baseline scores before treatments indicated the extent of QoL impairment.

## Methods

This study was based on consecutive patients who were treated due to localized T1-3N0M0 prostatic carcinoma with three-dimensional conformal radiotherapy in the years 2003-2007. Treatment plans were calculated using a four-field box technique with 15 MeV photons and a multileaf collimator, as reported recently in detail [17]. A margin of 1.5 cm in the anterior/lateral and 1 cm in the craniocaudal and dorsal directions to the CTV (prostate with or without seminal vesicles) was applied to define the PTV. The total dose to the prostate in the reference point was 70.2 or 72 Gy at 1.8 or 2.0 Gy daily fractions. The integral dose (AUC-area under the curve) was defined as the relation of the area under the dose-volume histogram curve to the total area, multiplied by 100.

An initial group of 324 patients has been surveyed prospectively before (time A), at the last day (B), two months (median, range 6 weeks-6 months, 71% within 9 weeks) after (C) and sixteen months (median, range 12-20 months) after (D) radiotherapy using a validated questionnaire, the Expanded Prostate Cancer Index Composite (EPIC) [18,19]. The questionnaire comprises 50 items concerning the urinary, bowel, sexual and hormonal domains for function and bothersomeness. Only patients with questionnaire results from both time A and time D have been included in the analysis (92% of the initial group), resulting in 298 (A), 213 (B), 267 (C) and 298 (D) questionnaires at the respective points in time. The multi-item scale scores were transformed lineary to a 0-100 scale, with higher scores representing better health-related quality of life (QoL). In accordance with data in the literature, mean QoL changes of below 5 points can be defined as clinically not significant, 5-10 as "little" changes, 10-20 as "moderate" changes and >20 as "very much" changes [20,21].

The questionnaire was handed over to the patients personally by one of the physicians at time A, B and C. Patients presented in the department six to ten weeks after the end of treatment. Missed questionnaires in the acute phase (time C) and questionnaires one to two years after radiotherapy (time D) were sent to the patients with a return envelope. If a questionnaire was not returned within four weeks, patients were contacted by telephone and urged to complete it.

Those 10% of patients who reported the greatest adverse *changes *of urinary or bowel bother scores (implicating 7 items, respectively) at time D were in a particular focus of this study. They were defined as patients with adverse long-term urinary or bowel QoL. To evaluate the impact of pretreatment scores on posttreatment scores, patients were divided into quartiles in dependence on their pretreatment urinary or bowel bother scores. Patients with the best pretreatment QoL were subsumed in an upper quartile (those 25% of patients with the highest scores), patients with the worst pretreatment QoL in a lower quartile (those 25% of patients with the highest scores), remaining patients in the medial quarters.

Sexual and hormonal domains were not considered in this evaluation. As previously demonstrated, sexual function does usually not recover after a decline in the acute phase [22], whereas the hormonal domain cannot be regarded as a domain with major effects from the local radiotherapy treatment.

Statistical analysis was performed using the SPSS 17.0 (SPSS, Chicago, Ill), software. The Wilcoxon's matched-pairs test was applied to determine longitudinal changes in specific subgroups of patients. To explore statistical QoL score differences between different subgroups at a specific time, the Mann-Whitney-U-test was used. Contingency table analysis with the chi-square test was performed to compare treatment groups with respect to categorical variables. To assess the correlation between different scores or score changes, Spearman's rho was determined (correlation coefficient >0.4 considered as a relevant correlation). In a forward stepwise univariate and multivariate analysis, pretreatment scores and score changes were tested for their impact on adverse long-term urinary or bowel QoL. All p-values reported are two-sided, p < 0.05 is considered significant.

## Results

The median patient age was 71 (45-84) years. Patients could be classified as low risk (PSA ≤ 10 ng/ml; Gleason score <7; clinical T-stage ≤ 2a), intermediate risk (PSA 10-20 ng/ml or Gleason score = 7 or clinical T-stage 2b-c) and high risk (two risk factors for intermediate risk or PSA > 20 ng/ml or Gleason score >7 or clinical T-stage >2c) patients in 37%, 35% and 28%, respectively.

**Figure 1:**
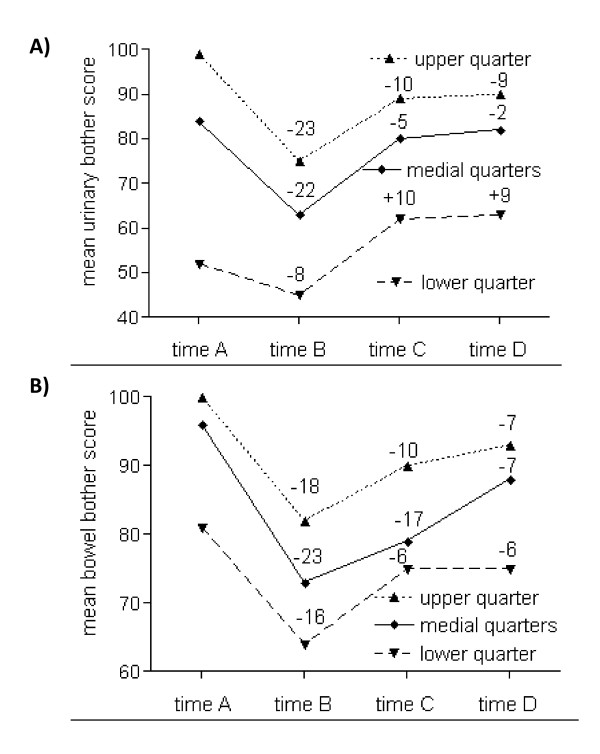
**Mean urinary (A) and bowel (B) bother scores in dependence on the baseline scores**. A: Baseline score for lower quarter: <70; medial quarter: 70-95; upper quarter: >95. All changes statistically significant (p < 0.05), except changes at time D for patients in the medial quarter. B: Baseline score for lower quarter: <93; medial quarter: 93-99; upper quarter: 100. All changes statistically significant (p < 0.05).

Focusing on the dependence of posttreatment scores from pretreatment scores, a different course of urinary and bowel bother scores could be clearly demonstrated (Figure 1). Patients with the worst - medial - best pretreatment urinary bother scores improved - remained stable - worsened significantly at times C and D. Scores at time C were nearly identical as the scores at time D. In the bowel domain, the scores improved significantly between times B and C in every subgroup, comparably to the urinary domain. A further improvement between times C and D was noticed in the subgroups with medial and best pretreatment bowel bother scores. Relative to baseline levels, mean scores of all subgroups decreased at time D in the range of 6-7 points.

**Figure 2:**
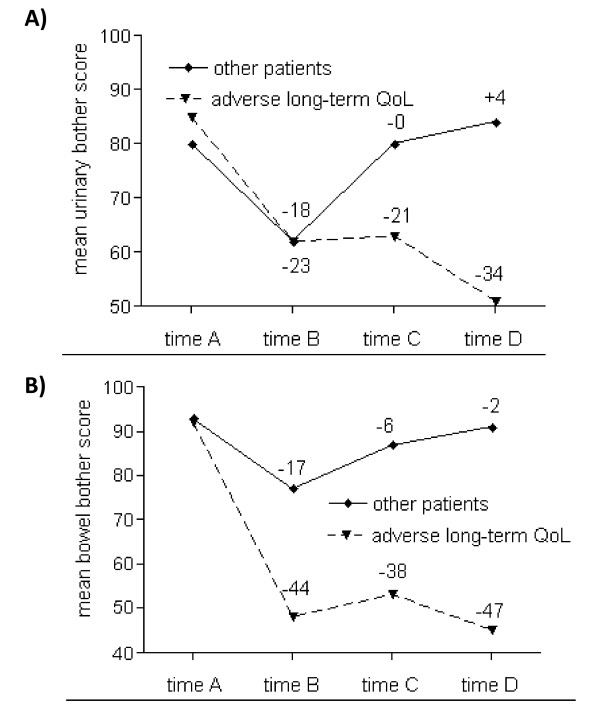
**Mean urinary (A) and bowel (B) bother scores for patients with vs. without adverse long-term urinary quality of life (QoL)**.

Treatment-related characteristics (fraction dose, total dose, prostate volume, PTV, AUC for bladder and rectum, organ volumes within any of the isodoses 10-100%, percentage of patients with neoadjuvant hormonal therapy) did not differ significantly for the patients who were selected as patients with adverse long-term urinary or bowel QoL in comparison to other patients (Table 1). Looking at the prior scores of these patients, only small differences could be seen before treatment (Figure 2). Urinary bother scores were diverging clearly at time C, in contrast to a divergence of bowel bother scores already at time B.

**Table 1:** Demographic and treatment-related characteristics of patients with vs. without adverse long-term quality of life (QoL) scores

	adverse long-term urinary QoL		adverse long-term bowel QoL	
	**yes (n = 261)**	**no (n = 35)**	**yes (n = 264)**	**no (n = 32)**

patient age/years median (range)	72 (51-83)	71 (45-84)	71 (51-82)	72 (45-84)

% fraction dose 1.8 Gy	69%	72%	72%	72%

% total dose 72 Gy	54%	56%	59%	55%

prostate volume/cc median (range)	40 (18-107)	39 (11-151)	36 (14-107)	40 (11-151)

% NHT	34%	32%	38%	32%

PTV/cc median (range)	338 (212-529)	330 (169-631)	339 (117-517)	330 (177-517)

bladder volume/cc median (range)	220 (54-657)	192 (14-806)	181 (82-657)	194 (14-806)

rectum volume/cc median (range)	102 (43-295)	97 (28-401)	93 (38-295)	98 (28-401)

AUC for bladder/% median (range)	37 (12-69)	41 (7-98)	35 (7-78)	41 (7-98)

bladder volume within 90% isodose*/%	20 (5-46)	21 (2-64)	20 (3-54)	22 (2-57)

AUC for rectum/% median (range)	52 (33-77)	51 (19-84)	47 (23-70)	52 (19-84)

rectum volume within 90% isodose*/%	27 (6-64)	28 (6-60)	24 (12-50)	28 (6-64)

*100% = prescription dose of 70.2-72 Gy; 90% = 63.2-64.8 Gy

Focusing on great/moderate bother from particular problems (specific items of the questionnaire), we found a missing dependence from pretreatment symptoms, and the strongest dependence from symptoms several weeks after radiotherapy (Table 2). Patients reporting great/moderate bother with urinary/bowel problems at time C reported to have great/moderate bother at time D in ≥ 50%, respectively. Only ≤ 7% of patients without great/moderate bother with urinary/bowel problems at time C reported to have great/moderate bother at time D.

**Table 2:** Percentage of patients reporting certain long-term bother (time D) in dependence on pretreatment (time A) or acute (time B and C) bother

	probability of long-term bother at time D if bother already present vs. absent at time A/B/C [p-value]		
**great/moderate bother with:**	**time A**	**time B**	**time C**

dripping or leaking urine	6% vs. 8% n.s.	19% vs. 3% [0.001]	50% vs. 3% [<0.001]

pain or burning on urination	0% vs. 6% n.s.	11% vs. 1% [0.001]	28% vs. 3% [<0.001]

waking up to urinate	26% vs. 29% n.s.	44% vs. 8% [<0.001]	72% vs. 13% [<0.001]

urinary function overall	7% vs. 16% n.s.	24% vs. 5% [<0.001]	50% vs. 7% [<0.001]

urgency to have a bowel movement	21% vs. 13% n.s.	25% vs. 5% [<0.001]	50% vs. 6% [<0.001]

losing control of stools	25% vs. 5% n.s.	25% vs. 1% [<0.001]	45% vs. 3% [<0.001]

bloody stools	- vs. 3%*	0% vs. 3% n.s.	33% vs. 2% [<0.001]

bowel habits overall	28% vs. 13% n.s.	34% vs. 3% [<0.001]	56% vs. 5% [<0.001]

Example: 56% of patients with great/moderate bother with bowel habits overall at time C report the same bother at time D; 5% of patients without great/moderate bother with bowel habits overall at time C report this bother at time D

n.s. = not significant

*no patients with great/moderate bother with bloody stools at time A

Defining bother score decreases >20 points as severe changes, patients with vs. without severe urinary QoL changes at times B and C were found to also have severe urinary QoL changes at time D in 14% vs. 6% (p = 0.042) and 40% vs. 7% (p < 0.001). Patients with vs. without severe bowel QoL changes at times B and C were found to also have severe bowel QoL changes at time D in 30% vs. 7% (p < 0.001) and 49% vs. 7% (p < 0.001).

Correlating absolute urinary and bowel scores at different intervals, good intra- and interdomain correlations of urinary and bowel scores became evident - with higher correlation indices after than before radiotherapy. A strong correlation between scores at different intervals within a specific domain resulted (Table 3). Considering pretreatment urinary/bowel QoL scores and QoL score changes relative to baseline scores at times B, C and D, the highest correlation coefficients were found between changes at times C and D (r > 0.5; p < 0.001), respectively (Table 4, pretreatment scores not shown due to low correlation coefficients r < 0.4).

**Table 3:** Spearman's correlation index between quality of life scores (only indices r > 0.4 shown; p < 0.001 for all)

	UBS time A	BBS time A	UBS time B	BBS time B	UBS time C	BBS time C	UBS time D	BBS time D
UBS time A		0.43	0.54	-	0.56	-	0.55	-

BBS time A	0.43		-	-	-	-	-	0.48

UBS time B	0.54	-		0.54	0.64	-	0.56	-

BBS time B	-	-	0.54		0.42	0.63	0.41	0.50

UBS time C	0.56	-	0.64	0.42		0.53	0.69	-

BBS time C	-	-	-	0.63	0.53		0.46	0.57

UBS time D	0.55	-	0.56	0.41	0.69	0.46		0.54

BBS time D	-	0.48	-	0.50	-	0.57	0.54	

*Abbreviations: *UBS = urinary bother score; BBS = bowel bother score

**Table 4:** Spearman's correlation index between quality of life score changes relative to baseline scores at time A (only indices r > 0.4 shown; p < 0.001 for all)

	UBS change time B	BBS change time B	UBS change time C	BBS change time C	UBS change time D	BBS change time D
UBS change time B		0.45	0.51	-	-	-

BBS change time B	0.45		-	0.53	-	-

UBS change time C	0.51	-		-	0.55	-

BBS change time C	-	0.53	-		-	0.52

*Abbreviations: *UBS = urinary bother score; BBS = bowel bother score

In a univariate analysis of factors for adverse long-term urinary and bowel QoL (Table 5), various factors were found to significantly predict adverse long-term QoL - including crossover relations between the urinary and bowel domains. Patients with adverse long-term urinary QoL were more likely to have adverse long-term bowel QoL and vice versa (patients with vs. without adverse urinary QoL reported a mean bowel score decrease of 21 vs. 5 points at time D; p < 0.001; patients with vs. without adverse bowel QoL reported a mean urinary score decrease of 16 vs. -1 points at time D; p < 0.001).

**Table 5:** Predictive factors for low long-term quality of life (QoL) in univariate and multivariate analysis (urinary and bowel bother scores at time A and score changes relative to baseline scores at times B and C were tested; significant factors are presented)

		univariate analysis		multivariate analysis	
**item**	**risk factor**	**hazard ratio** **[95% CI]**	**p-value**	**hazard ratio** **[95% CI]**	**p-value**

low long-term urinary QoL	urinary bother score at time B	1.03 [1.00-1.05]	0.032	-	-
	
	urinary bother score change at time C	1.06 [1.04-1.08]	<0.001	1.06 [1.04-1.08]	<0.001
	
	bowel bother score at time A	1.03 [1.00-1.05]	0.028	-	-
	
	bowel bother score change at time C	1.03 [1.01-1.04]	0.002	-	-

low long-term bowel QoL	urinary bother score change at time B	1.02 [1.00-1.04]	0.048	-	-
	
	urinary bother score change at time C	1.02 [1.00-1.04]	0.031	-	-
	
	bowel bother score change at time B	1.05 [1.03-1.08]	<0.001	1.03 [1.00-1.06]	0.049
	
	bowel bother score change at time C	1.06 [1.04-1.08]	<0.001	1.05 [1.02-1.08]	<0.001

The multivariate analysis was performed to demonstrate independent factors - intradomain score changes relative to baseline at time C proved to be independent significant predictors for adverse score changes at time D, respectively. Additionally, bowel QoL changes at time B were independently predictive for adverse long-term bowel QoL (Table 5).

## Discussion

In this study, we could demonstrate the strong influence of acute side effects on long-term toxicity in prostate cancer treatment. In contrast to studies in the past, based on a grading system [12,13], a quality of life analysis was used to elaborate the impact of consequential late effects on long-term quality of life. EPIC questionnaire measurements have the advantage of being more sensitive to changes in acute bowel toxicity in comparison to RTOG acute morbidity scoring criteria or proctoscopic toxicity scores [23]. Apparently, prostate cancer radiotherapy with doses >70 Gy can lead to a relevant severity and duration of acute radiation effects. The intestinal or bladder mucosa is damaged to a considerable degree, so that an adequate barrier against mechanical and/or chemical stress is not present any more for a considerable period of time. The non-healing response can progress directly into a late effect. In contrast to a low percentage of patients (5-7%) who assess their urinary function or bowel habits to be a great or moderate problem more than a year after radiotherapy without the same assessment already several weeks after radiotherapy, ≥ 50% who reported one of these problems several weeks after radiotherapy still had the same bother more than a year after radiotherapy, respectively.

Urinary and bowel QoL after radiotherapy was found to be strongly dependent on urinary and bowel QoL before radiotherapy. Nevertheless, a difference was found between urinary and bowel QoL. Acute bowel problems were gradually improving over time. In comparison to baseline, scores at time D decreased 6-7 points for all subgroups. In contrast to bowel bother scores, no further improvement was noticed for urinary bother scores between time C and D. Significantly lower scores at times C and D in comparison to baseline were only found for the patients with the best baseline scores. Urinary QoL for the patients with initially very low scores improved significantly, suggesting a possible effect of radiotherapy on the reduction of benign prostatic hyperplasia. Long-term urinary and bowel scores at time D were correlating well with the respective scores at time A - but also B and C. Correlation indices were gradually improving over time, so that the best correlation was found between the respective scores at times C and D (Table 3).

A different aspect (main aspect) of this study is the evaluation of QoL score *changes *relative to baseline scores before treatment. In contrast to an absolute QoL level, adverse changes imply radiotherapy toxicity - impossible to assess with a single measurement after radiotherapy. In contrast to absolute scores, QoL score changes were not correlating with pretreatment scores. In respect of long-term scores, the best predictive value (greatest correlation index) was found for scores at time C, i.e. in case of considerable acute changes several weeks after treatment, comparable changes can still be expected more than one year after treatment.

Finally, we have focused on the patients with the greatest long-term QoL impairment relative to baseline scores. We could exclude a significant impact of treatment-related characteristics on this impairment for this particular patient group - not implying that these characteristics do not have any meaning for radiotherapy-related toxicity. As reported in the already published studies, factors like bladder volume, prostate volume or hormonal therapy have certainly an influence on particular problems [4,21]. A particular aspect of this evaluation is a homogenous treatment of the total study group concerning the technique, planning target volume definition and dose prescription. In most other study populations, patients with various techniques and total doses are combined [1,3,12,13,24]. The significant impact of dose to critical structures on toxicity could be demonstrated in these studies with different dose levels. This correlation could not be shown in our homogenous study population (all patients treated with the same technique to a dose of 70.2-72 Gy). We have to be aware that the dose-volume histogram is related to a single treatment planning CT scan. Taking into account changing organ volumes during the treatment [25,26], it might not be sensitive enough to discriminate clearly between patients with higher or lower volumes within certain dose levels over the entire treatment (all individual fractions).

Considering QoL scores of patients with the greatest long-term impairment in comparison to other patients (Figure 2), differences of QoL scores became well evident with time. A considerable divergence of urinary scores resulted at time C: patients with adverse long-term QoL were not able to recover from their acute symptoms - in contrast to a complete recovery for other patients. The impact of consequential late effects is demonstrated clearly in these curves. Patients with consequential late effects were not able to repair the acute tissue damage and QoL decreased even more with time. In contrast to the urinary domain, a drastic divergence of bowel bother scores resulted already at time B. A similar progression followed: patients with adverse long-term QoL were not able to repair the damage, while other patients (nearly) returned to their baseline levels before radiotherapy. The multivariate analysis supports well the results of these curves: urinary bother score changes at time C were highly predictive of adverse long-term urinary QoL; bowel bother score changes at times B and C were independently predictive for adverse long-term bowel QoL.

Urinary and bowel score changes at time C have been shown to predict both adverse urinary and bowel long-term QoL in univariate analysis. The interdomain predictions were not independent from the respective other domain, i.e. they were not independent factors in the multivariate analysis. An individual radiosensitivity is suggested by these data: patients with reduced repair capacity of the bladder wall or urethra are more likely to have a reduced repair capacity of the rectal wall and vice versa.

The results of this study emphasize the need of close follow-up and early prophylactic actions for patients with greater and longer acute radiotherapy-associated toxicities to possibly prevent late toxicities, though these possibilities are currently limited. The time to filter candidates for these actions can be two months after radiotherapy (median time of time C questionnaire) concerning urinary problems and the end of radiotherapy concerning bowel problems. Taking into account the well known association of dose with late toxicity [8,10], stopping the radiation treatment at a lower dose (for example 70 Gy instead of 80 Gy) might be advisable for selected patients (for example older low-risk patients with limited life expectancy and only questionable benefit of a dose escalation) with heavy acute bowel toxicity. This concept is currently not accepted. The decision for a dose prescription is independent from the severity of acute effects.

There are no standard prophylactic regimens to ameliorate urinary symptoms after external beam radiotherapy. After prostate brachytherapy - known to be associated with greater urinary morbidity in comparison to external beam radiotherapy - alpha-blockers are commonly administered. The beneficial effect on urinary symptoms has been shown in a placebo-controlled randomized study [27]. An advantage might also be possible for patients after external beam radiotherapy.

Patients with greater and longer acute rectal morbidity should be advised to avoid unnecessary mechanical rectal wall irritation - a low residue diet, reducing the frequency and volume of stools might be beneficial in this context. Constipation should be treated with adequate dietary measures or laxatives.

Medical treatment with anti-inflammatory drugs, like corticosteroids or mesalamine, can reduce acute inflammatory symptoms. However, a long-term effect could not be demonstrated in the past [28]. Anti-inflammatory drugs are inhibiting protein synthesis, so that tissue repair might even be impaired. Other drugs, like retinal palmitate (vitamin A) might more effectively promote wound healing [29]. Patients with acute bowel problems should in any case be specifically informed to avoid invasive procedures and biopsies of the rectal wall. Biopsy may cause persistent inflammation, decrease healing, and precipitate fistula formation [30].

## Conclusions

In contrast to absolute scores after radiotherapy, quality of life *changes *cannot be predicted by pretreatment scores. Consequential late effects play a major role after radiotherapy for prostate cancer. Long-term gastrointestinal symptoms are well predicted by symptoms at the end of and several weeks after treatment, suggesting an inefficiency of the repair system and a non-healing acute response. Urinary symptoms without recovery within a few weeks after radiotherapy are likewise highly predictive for adverse long-term urinary quality of life.

Patients with greater and longer acute toxicity are candidates for closer follow-up and possible prophylactic actions to reduce a high probability of long-term problems, including possibly a total dose reduction for selected patients with particularly bothersome acute gastrointestinal problems.

## Competing interests

The authors declare that they have no competing interests.

## Authors' contributions

MP, MJE have made substantial contributions to conception and design; MP and KF have made substantial contributions to acquisition of data; MP, RH, MDP, KF, SS, DS, MJE to analysis and interpretation of data. MP has been involved in drafting the manuscript. RH, MDP, KF, SS, DS, MJE revised it critically for important intellectual content. All authors have given final approval of the version to be published.

## References

[B1] Welz S Nyazi M Belka C Ganswindt U Surgery vs. radiotherapy in localized prostate cancer Radiat Oncol 2008 3 23 10.1186/1748-717X-3-23 18775078 PMC2553076

[B2] Cheung R Tucker SL Ye JS Dong L Liu H Huang E Mohan R Kuban D Characterization of rectal normal tissue complication probability after high-dose external beam radiotherapy for prostate cancer Int J Radiat Oncol Biol Phys 2004 58 1513 1519 10.1016/j.ijrobp.2003.09.015 15050331

[B3] Zapatero A Garcia-Vicente F Modolell I Alcantara P Floriano A Cruz-Conde A Torres JJ Pérez-Torrubia A Impact of mean rectal dose on late rectal bleeding after conformal radiotherapy for prostate cancer: dose-volume effect Int J Radiat Oncol Biol Phys 2004 59 1343 1351 15275719 10.1016/j.ijrobp.2004.01.031

[B4] Selek U Cheung R Lii M Allen P Steadham RE Vantreese TR Jr Little DJ Rosen II Kuban D Erectile dysfunction and radiation dose to penile base structures: a lack of correlation Int J Radiat Oncol Biol Phys 2004 59 1039 1046 10.1016/j.ijrobp.2003.12.028 15234037

[B5] Pinkawa M Fischedick K Asadpour B Gagel B Piroth MD Eble MJ Low-grade toxicity after conformal radiation therapy for prostate cancer - impact of bladder volume Int J Radiat Oncol Biol Phys 2006 64 835 841 16289911 10.1016/j.ijrobp.2005.09.003

[B6] Michalski JM Gay H Jackson A Tucker SL Deasy JO Radiation dose-volume effects in radiation-induced rectal injury Int J Radiat Oncol Biol Phys 2010 76 S123 S129 20171506 10.1016/j.ijrobp.2009.03.078 PMC3319467

[B7] Beckendorf V Guerif S Le Prise E Cosset J Bougnoux A Chauvet B Salem N Romenstaing P Luporsi I Bey P 70 Gy versus 80 Gy dose escalation getug 06 french trial for localized prostate cancer: mature results Int J Radiat Oncol Biol Phys 2008 72 S96 S97

[B8] Kuban DA Tucker SL Dong L Starkschall G Huang EH Cheung MR Lee AK Pollack A Long-term results of the M.D. Anderson randomized dose-escalation trial for prostate cancer Int J Radiat Oncol Biol Phys 2008 70 67 74 17765406 10.1016/j.ijrobp.2007.06.054

[B9] Peeters ST Heemsbergen WD Koper PC van Putten WL Slot A Dielwart MF Dielwart MFH Bonfer JMG Incrocci L Lebesque JV Dose-response in radiotherapy for localized prostate cancer: results of the Dutch multicenter randomized phase III trial comparing 68 Gy of radiotherapy with 78 Gy J Clin Oncol 2006 24 1990 1996 10.1200/JCO.2005.05.2530 16648499

[B10] Al-Mamgani A van Putten WL Heemsbergen WD van Leenders GJLH Slot A Dielwart MFH Incrocci L Lebesque JV Update of dutch multicenter dose-escalation trial of radiotherapy for localized prostate cancer Int J Radiat Oncol Biol Phys 2008 72 980 988 18495377 10.1016/j.ijrobp.2008.02.073

[B11] Dörr W Hendry JH Consequential late effects in normal tissues Radiother Oncol 2001 61 223 231 10.1016/S0167-8140(01)00429-7 11730991

[B12] Schultheiss TE Lee WR Hunt MA Hanlon AL Peter RS Hanks GE Late GI and GU complications in the treatment of prostate cancer Int J Radiat Oncol Biol Phys 1997 37 3 11 9054871 10.1016/s0360-3016(96)00468-3

[B13] Heemsbergen WD Peeters ST Koper PC Hoogeman MS Lebesque JV Acute and late gastrointestinal toxicity after radiotherapy in prostate cancer patients: consequential late damage Int J Radiat Oncol Biol Phys 2006 66 3 10 16814954 10.1016/j.ijrobp.2006.03.055

[B14] Joseph KJ Alvi R Skarsgard D Tonita J Pervez N Small C Tai P Analysis of health-related quality of life (HRQoL) of patients with clinically localized prostate cancer, one year after treatment with external beam radiotherapy (EBRT) alone versus EBRT and high dose rate brachytherapy (HDRBT) Radiat Oncol 2008 3 20 10.1186/1748-717X-3-20 18627617 PMC2494997

[B15] Fransson P Lund J Damber J Klepp O Wiklund F Fossa S Widmark A Quality of life in patients with locally advanced prostate cancer given endocrine treatment with or without radiotherapy; 4-year follow-up of SPCG-7/SFUO-3, an open-label, randomised, phase III trial Lancet Oncol 2009 10 370 380 10.1016/S1470-2045(09)70027-0 19286422

[B16] Geinitz H Thamm R Scholz C Heinrich C Prause N Kerndl S Keller M Busch R Molls M Zimmermann FB Longitudinal analysis of quality of life in patients receiving conformal radiation therapy for prostate cancer Strahlenther Onkol 2010 186 46 52 10.1007/s00066-009-2023-7 20082188

[B17] Pinkawa M Piroth MD Fischedick K Nussen S Klotz J Holy R Eble MJ Self-assessed toxicity after external beam radiotherapy for prostate cancer - predictive factors on irritative symptoms, incontinence and rectal bleeding Radiat Oncol 2009 4 36 10.1186/1748-717X-4-36 19772568 PMC2753361

[B18] Wei JT Dunn RL Litwin MS Sandler HM Sanda MG Development and validation of the expanded prostate cancer index composite (epic) for comprehensive assessment of health-related quality of life in men with prostate cancer Urology 2000 56 899 905 10.1016/S0090-4295(00)00858-X 11113727

[B19] Volz-Sidiropoulou E Pinkawa M Fischedick K Jakse G Gauggel S Eble MJ Factor analysis of the Expanded Prostate Cancer Index Composite (EPIC) in a patient group after primary (external beam radiotherapy and permanent iodine-125 brachytherapy) and postoperative radiotherapy for prostate cancer Curr Urol 2008 2 122 129 10.1159/000189652

[B20] Ososba D Rodrigues G Myles J Zee B Pater J Interpreting the significance of changes in health-related quality-of-life scores J Clin Oncol 1998 16 139 144 9440735 10.1200/JCO.1998.16.1.139

[B21] Pinkawa M Fischedick K Asadpour B Gagel B Piroth MD Nussen S Eble MJ Toxicity profile with a large prostate volume after external beam radiotherapy for localized prostate cancer Int J Radiat Oncol Biol Phys 2008 70 83 89 17855010 10.1016/j.ijrobp.2007.05.051

[B22] Pinkawa M Gagel B Piroth MD Fischedick K Asadpour B Kehl M Klotz J Eble MJ Erectile dysfunction after external beam radiotherapy for prostate cancer Eur Urol 2009 55 227 236 10.1016/j.eururo.2008.03.026 18375048

[B23] Muanza TM Albert PS Smith S Godette D Crouse NS Cooley-Zgela T Scinto L Camphausen K Coleman CN Ménard C Comparing measures of acute bowel toxicity in patients with prostate cancer treated with external beam radiation therapy Int J Radiat Oncol Biol Phys 2005 62 1316 1321 16029787 10.1016/j.ijrobp.2004.12.083

[B24] Michalski JM Winter K Purdy JA Parliament M Wong H Perez CA Roach M Bosch W Cox JD Toxicity after three-dimensional radiotherapy for prostate cancer on RTOG 9406 dose Level V Int J Radiat Oncol Biol Phys 2005 62 706 713 15936549 10.1016/j.ijrobp.2004.11.028

[B25] Martin JM Bayley A Bristow R Chung P Gospodarowicz M Menard C Milosevic M Rosewall T Wade PR Catton CN Image guided dose escalated prostate radiotherapy: still room to improve Radiat Oncol 2008 4 50 10.1186/1748-717X-4-50 PMC2777178 19887007

[B26] Pinkawa M Pursch-Lee M Asadpour B Gagel B Piroth MD Nussen S Eble MJ Image-guided radiotherapy for prostate cancer. Implementation of ultrasound-based prostate localization for the analysis of inter- and intrafractiuon organ motion Strahlenther Onkol 2008 184 679 685 10.1007/s00066-008-1902-7 19107350

[B27] Elshaikh MA Ulchaker JC Reddy CA Angermeier KW Klein EA Chehade N Altman A Ciezki JP Prophylactic tamsulosin (Flomax) in patients undergoing prostate 125I brachytherapy for prostate carcinoma: final report of a double-blind placebo-controlled randomized study Int J Radiat Oncol Biol Phys 2005 62 164 169 10.1016/j.ijrobp.2004.09.036 15850917

[B28] Fantin AC Binek J Jabar FM Meyenberger C Argon beam coagulation for treatment of symptomatic radiation-induced proctitis Gastrointest Endosc 1999 49 515 518 10.1016/S0016-5107(99)70054-4 10202070

[B29] Ehrenpreis ED Jani A Levitsky J Ahn J Hong J A prospective, randomized, double-blind, placebo-controlled trial of retinol-palmitate (vitamin A) for symptomatic chronic radiation proctopathy Dis Colon Rectum 2005 48 1 8 10.1007/s10350-004-0821-7 15690650

[B30] Tran A Wallner K Merrick GS Seeberger J Armstrong J Mueller A Cavanagh W Lin D Butler W Rectal fistulas following prostate brachytherapy Int J Radiat Oncol Biol Phys 2005 63 150 154 16111583 10.1016/j.ijrobp.2005.01.021

